# Rehabilitation of patients after COVID-19 recovery: An experience at the Physical and Rehabilitation Medicine Institute and Lucy Montoro Rehabilitation Institute

**DOI:** 10.6061/clinics/2021/e2804

**Published:** 2021-06-07

**Authors:** Marta Imamura, Aline Rossetti Mirisola, Fernando de Quadros Ribeiro, Lucas Ramos De Pretto, Fábio Marcon Alfieri, Vinicius Ramos Delgado, Linamara Rizzo Battistella

**Affiliations:** I Faculdade de Medicina FMUSP, Universidade de Sao Paulo, Sao Paulo, SP, BR; II Instituto de Medicina Fisica e Reabilitacao, Hospital das Clinicas HCFMUSP, Faculdade de Medicina, Universidade de Sao Paulo, Sao Paulo, SP, BR; III Centro de Pesquisa Clinica, Instituto de Medicina Fisica e Reabilitacao, Hospital das Clinicas HCFMUSP, Faculdade de Medicina, Universidade de Sao Paulo, Sao Paulo, SP, BR; IV Centro de Lasers e Aplicacoes, Instituto de Pesquisas Energeticas e Nucleares (IPEN-CNEN/SP), Sao Paulo, SP, BR

**Keywords:** Rehabilitation, Rehabilitation Center, Treatment Outcome, Rehabilitation Services, Inpatient Rehabilitation, Coronavirus Infection

## Abstract

**OBJECTIVES:**

As patients recovering from the novel coronavirus disease 2019 (COVID-19) present with physical, respiratory, cognitive, nutritional, and swallowing-related impairments and mental health complications, their rehabilitation needs are complex. This study aimed to describe the demographic, clinical, and functional status after the discharge of COVID-19 survivors who underwent intensive multidisciplinary inpatient rehabilitation at the Physical and Rehabilitation Medicine Institute of the University of Sao Paulo Medical School General Hospital and Lucy Montoro Rehabilitation Institute. We determined the most important factors related to the length of inpatient rehabilitation treatment and present the functional outcomes.

**METHODS:**

This was a retrospective study based on electronic medical records. In addition to the severity of COVID-19 and length of hospital stay for the management of COVID-19 and comorbidities, we collected sociodemographic data including age, sex, height, and weight. Functional assessments were performed using the Functional Independence Measure (FIM); Short Physical Performance Battery; Montreal Cognitive Assessment; Depression, Anxiety and Stress Scale; Revised Impact of Events Scale; bioelectrical impedance; Functional Oral Intake Scale; oropharyngeal dysphagia classification; and nutritional assessment.

**RESULTS:**

There was a significant improvement in FIM before and after inpatient rehabilitation treatment (*p<*0.0001). Muscle strength and walking capacity were significantly improved (*p<*0.01). The most important factors related to the length of inpatient rehabilitation treatment were improvement in FIM scores (Spearman’s r=0.71) and gain in lean mass (Spearman’s r=0.79).

**CONCLUSIONS:**

Rehabilitation of patients after COVID-19 recovery improves their functional status and should be considered in the post-acute phase for selected patients with COVID-19.

## INTRODUCTION

Rehabilitation is a key strategy for public health in the 21^st^ century. Prior to the coronavirus disease 2019 (COVID-19) pandemic, it was expected that one in every three people would require rehabilitation due to illness or injuries worldwide (1). The estimated number of 2.45 billion people in need of rehabilitation (1) is further challenged by the emerging physical, mental, cognitive, neurological, nutritional, swallowing-related, and respiratory complications identified in COVID-19 survivors (2-4). Brazil is a global COVID-19 hotspot, with a high number of cases and deaths, as shown by the World Health Organization’s Health Emergency Dashboard on the disease (5). It should be noted that the Brazilian population is at risk of developing severe COVID-19 due to the prevalence of factors such as old age and comorbidities, among others. However, as a country of continental dimensions, populations have different profiles in each region, as well as different social behaviors, genetic characteristics, and socioeconomic backgrounds, requiring diverse measures to contain the infection in each region (6).

The impact of COVID-19 on the continuity of health services and the burden on health professionals worldwide has been well documented (7). Comprehensive rehabilitation after COVID-19 recovery requires environmental changes for professionals, patients, and their families. The status of rehabilitation services in 12 countries during the outbreak has been described elsewhere, reinforcing the need to improve rehabilitation service delivery strategies in the face of the pandemic (7).

Inpatient, outpatient, and community-delivered services are recommended for the rehabilitation of COVID-19 survivors, based on their individual needs (3,4,8-10). It is important to emphasize that in addition to respiratory manifestations (3), neurological and psychiatric complications, and decline in physical, cognitive, and psychosocial functions, together with the complications of intensive care and hospitalization necessitate further rehabilitation in COVID-19 survivors (4,8-10). Innovative strategies including virtual rehabilitation and prehabilitation are also described in the context of the COVID-19 pandemic (9). In light of this, rehabilitation facilities began providing services along the continuum of care, including providing service within the intensive care units (ICUs), developing service delivery strategies and specialized interventions, and adapting treatment settings to meet the needs of these patients (4,9,10). The first systematic review on rehabilitation needs arising from COVID-19 indicated that early rehabilitation should be offered during the acute phase; people affected by social distancing measures should receive guidance on exercise routines to reduce the risk of muscle weakness, frailty, depression, and cognitive complications (11). Tele-rehabilitation strategies have the potential to reach people in their homes (11). As recommended by the WHO, where rehabilitation needs are identified, patients should be referred appropriately (8).

São Paulo is the wealthiest state in Brazil and is located in the southeastern region of the country. The Physical and Rehabilitation Medicine Institute of the University of Sao Paulo Medical School General Hospital (IMREA) and the Lucy Montoro Rehabilitation Institute (IRLM), are based in the state capital city, and provide comprehensive, multidisciplinary inpatient and outpatient rehabilitation services, including outreach services. These facilities provide specialized rehabilitation services to people with disabling health conditions that require diverse treatment strategies. For example, intensive inpatient rehabilitation has already shown positive motor and cognitive outcomes in individuals with stroke, brain and spinal cord injuries, and Guillain-Barré Syndrome. In 2014, IMREA and IRLM were the first Brazilian institutions certified by the Commission on Accreditation of Rehabilitation Facilities. As such, they value investigating and continuously improving the outcomes of the services provided. Therefore, it was necessary to follow up on the results and investigate treatment outcomes in COVID-19 survivors.

Based on the expertise in delivering intensive inpatient rehabilitation services for persons with various health conditions, and in view of the need to offer rehabilitation care for COVID-19 survivors, IMREA and IRLM adapted their models of care to cope with the situation. Moreover, the gap in scientific evidence on rehabilitation treatments for patients with COVID-19 along treatment phases and the continuum of care strengthened the case for this study.

Our objective was to describe the demographic, clinical, and functional status of COVID-19 survivors who underwent comprehensive, intensive, individualized, multidisciplinary inpatient rehabilitation at IMREA or IRLM, after their discharge from the hospital. Further, we aimed to present functional rehabilitation outcomes in terms of the Functional Independence Measure (FIM) scores and determine the most important factors related to the duration of inpatient rehabilitation.

## MATERIALS AND METHODS

### Patients

This study was a retrospective case series without a control group. We retrieved data of patients with a clinical diagnosis of COVID-19, laboratory confirmed severe acute respiratory syndrome coronavirus 2 (SARS-CoV-2) infection (by either polymerase chain reaction [PCR] or serology testing), and who received intensive inpatient rehabilitation treatment at IMREA or IRLM between April 14 and September 14, 2020.

The inclusion criteria for inpatient rehabilitation were as follows: age>14 years; PCR-confirmed SARS-CoV-2 infection or confirmed diagnosis of COVID-19 by serology with symptoms for at least 28 days; and absence of active symptoms of flu syndrome for at least 3 days with at least one negative PCR report for SARS-CoV-2.

The exclusion criteria were as follows: fever for at least 3 days, patients requiring antipyretic medications, current mechanical ventilation, chronic kidney disease requiring dialysis, pressure ulcers with indication of surgical treatment, alternative feeding routes, current treatment for cancer or immunotherapeutic treatments, immunosuppression, clinical instability, unstable mental illness, or active drug addiction.

All patients were admitted from tertiary hospitals within the transition of care therapeutic approach in the sub-acute phase of the disease, with a relative or legal guardian acting as a caregiver during the inpatient treatment.

### Ethics

The study was approved by the Institutional Review Board of the University of Sao Paulo Medical School General Hospital (CAPPesq: Comissão de Ética para Análise de Projetos de Pesquisa do HCFMUSP), with approval number CAEE 38637620.8.0000.0068.

## METHODS

IMREA and IRLM offered comprehensive, intensive, individualized multidisciplinary inpatient rehabilitation to COVID-19 survivors. The treatment comprised systematic assessments that guided the weekly training routines delivered by physical, occupational, and speech therapists, and physical fitness, psychology, nutrition and dietetics, nursing, social services, and medical teams. This target-oriented, individualized routine included two to six 50-minute long sessions with each of these specialized teams, 5 to 7 days a week. Our framework and timeline for the disease was based on the WHO guidance on the post-COVID-19 conditions, which relates to the treatment of the acute phase of infection with hospitalization or acute illness in patients who have not been admitted to hospitals, post-acute phase with the initial 4 to 8 weeks after hospital discharge, and long-term follow-up with continued visits at 3-month intervals for people with persistent and chronic symptoms.

### Data collection

Sociodemographic and clinical information retrieved from electronic medical records included age, sex, height, weight, body mass index (BMI), length of hospital stay for the management of COVID-19 (in COVID-19 wards and/or ICU), classification of the COVID-19 severity level using the WHO standard (mild, moderate, severe, or critical) (8), duration since hospital discharge, presence of comorbidities (hypertension, diabetes, obesity, heart disease, vascular disease, asthma, and cancer), and the modified Charlson comorbidity index.

The retrieved data also included the results of other routine clinical evaluations during rehabilitation at these facilities, which included all motor and cognitive components of FIM (12). For the respiratory assessment, we measured the maximum inspiratory and expiratory pressures using a manovacuometer. The results were adjusted for age and sex according to previously reported guidelines (13).

Muscle strength was assessed using the Medical Research Council sum score (MRC) (14), ranging from 0 to 60. MRC values <48 correlate with muscle weakness and are considered severe when <36 (14). The functional ambulation categories (FAC) assessed gait capacity on a 6-point Likert scale (0 to 5) and determined the assistive devices required (15). The short physical performance battery (16) comprised the assessment of static body balance and ability to walk and stand up from a chair. We evaluated individuals’ balance at normal standing, semi-tandem, and tandem positions; time to walk four meters; and time to rise from a chair with arms in front of the body and return to the seated position five times.

The Montreal Cognitive Assessment screens many domains, including executive functions, visual-spatial abilities, memory, attention, concentration, working memory, language, and time orientation, with individuals scoring up to 30 points (17). We considered a cut-off score of 26 to identify cognitive impairment (18). The Depression, Anxiety and Stress Scale (DASS-21) uses a self-reported Likert-like scale that covers 21 mental health-related items in the week before the test (19). Normal scores were determined for the depression (0 to 9), anxiety (0 to 6), and stress (0 to 10) subscales. The Revised Impact of Events Scale, a 22-item questionnaire, was used to identify symptoms of post-traumatic stress disorder, with regard to all three components of the scale: avoidance, intrusion, and hyperarousal (20). The normal scores ranged from 0 to 23 (20).

We used bioelectrical impedance (Biodynamics^®^ model 310e^®^) to measure body composition, as it is a sensitive, fast, safe, and radiation-free test (21). Our measures included the percentages of fat and lean mass. The Functional Oral Intake Scale was also chosen for its ease of use and short application time, enabling testers to quickly identify any issues with oral intake (22). The oropharyngeal dysphagia classification (23) was used to provide further details on swallowing difficulties. Nutritional status, history, and risks were assessed based on the medical records, as registered by the local nutrition and dietetics services staff, and the lean body mass was measured using the measurement of the triceps skinfold and arm and calf circumferences. We categorized the severity of malnutrition as absent, moderate, or severe according to the Global Leadership Initiative for Malnutrition (GLIM) (24).

### Rehabilitation program

General precautions recommended during the COVID-19 pandemic were in place in both rehabilitation facilities. Rehabilitation treatment was coordinated by a physical and rehabilitation medicine specialist. Medical staff ensured proper oxygen levels and maintenance of vital signs in the patients during therapies, managed comorbidities, and prescribed medications for management of anxiety and depressive symptoms, as well as cognitive stimulation and pain management, as needed. Nursing sessions aimed to prevent, monitor, and perform early diagnosis of aspiration pneumonia; administer drugs; and educate patients to increase adherence to medications after discharge. The nursing team also monitored vital signs three times a day, and supported self-care activities and changes in posture, whenever needed. Posture changes were performed every 2h for bedridden patients. Nurses also collected samples for laboratory monitoring tests, classified and managed the risk of falls, and monitored and managed skin integrity and bladder functions.

Joint sessions for respiratory physiotherapy and speech therapy included assistance with diaphragmatic breathing, changing postures for secretion drainage, resistive breathing exercises, assistance with resting respiratory muscles, conducting coordination exercises for breathing, speech and articulation, and vocal exercises. The inpatient routine included bedside visits and exercising three times a week, including monitoring during meals to update food consistency guidelines.

Physical therapy teams administered a conventional protocol of stretching, muscle strengthening, mobilization, functional and resistance training, including active cycle ergometer activities for the lower limbs; functional electrical stimulation-assisted training; sensory stimulation; orthostatic positioning; balance, gait, and body awareness training; and safety guidance for performing activities of daily living independently. Additionally, according to individual prescriptions, patients also received robot-assisted and virtual reality-assisted rehabilitation. Cycle ergometer and conditioning training were performed twice or thrice a week, according to established heartbeat and blood pressure limits for rest, stress, and therapeutic activities. Training intensity was monitored and adjusted based on the Borg rating of perceived exertion.

During the occupational therapy sessions, patients were assessed for their performance in self-care activities. Local and instrumental adaptations for the performance of daily activities and energy conservation techniques were provided during the four occupational therapy sessions per week. Psychological approaches, including relaxation maneuvers, biofeedback, and cognitive behavioral interventions to reduce tension were provided as needed. Speech therapy included a comprehensive approach for swallowing, and food and liquid consistencies were adjusted according to patients’ needs.

Malnutrition was managed by proper protein and calorie intake. In addition to body composition analysis, comprehensive nutritional assessment also covered food preferences, presence of permanent or temporary dietary restrictions, eating habits, and indication of protein or energy supplementation. Daily monitoring of adherence to the dietary plan was facilitated by the inpatient program. Individualized counseling and education on healthy eating habits and the preparation of suggested menus to increase diet therapy adherence after discharge was also included in the program. Social workers assisted in the transition of care after discharge.

Individual status at discharge was classified as “goals fully met,” “goals partially met,” or “goals unmet,” according to the assessment of the rehabilitation team about individuals’ treatment goals and outcomes achieved.

To lower the risk of bias and increase impartiality, data analyses were conducted by researchers who did not have access to the participants or the teams responsible for conducting assessments and treatment of the patients.

### Statistical analysis

All analyses were performed using Python and open-source libraries (25). Where applicable, continuous variables are presented as mean±standard deviation or range, while categorical data are presented as count (%).

Given the low number of patients studied, we opted for ranked, non-parametric tests in our analysis to avoid relying on a normal distribution of our dataset. To check for differences between group means, we chose the Kruskal-Wallis test by ranks and the Holm-Bonferroni correction for multiple tests. Statistical significance was set at *p*<0.05.

We created variables representing the evolution of scores after treatment. These variables were called “Delta” and were defined as the difference between the value at discharge from the rehabilitation center and the corresponding value at admission. For the ordinal (categorical) variables, we analyzed the number of orders of difference between the initial and final results for each patient, that is, the difference in the ordinal categories for each patient. To test for correlation between the treatment duration and the “Delta” variables, we used Spearman’s rank-order correlation.

## RESULTS

This study included 27 patients, of which, 23 fully achieved the functioning goals at discharge, while four patients were discharged at their own request, with the goals partially met. Therefore, the full clinical information on comorbidities, COVID-19 symptoms, and hospitalization during the acute phase was available for 23 of the 27 patients. All 23 patients (100%) had a period of ICU hospitalization. The mean length of stay in the COVID-19 ward was approximately 45 days (44.96±23.00 days), while the mean length of stay in ICU was approximately 30 days (30.04±18.23 days). Thus, the mean value of the total length of hospital stay was 75±40.46 days. Complete information regarding hospitalization is presented in Table 1. Furthermore, all patients (n=23, 100%) required endotracheal intubation during hospitalization. The mean duration of intubation was 22.61±14.33 days (95% confidence interval [CI]: 15.59-29.64 days). Thirteen patients (55.62%; 95% CI: 36.81%-74.37%) also required dialysis. The mean duration of dialysis treatment was 16.67±12.29 days (95% CI: 9.31-24.03 days). Additionally, 17 patients received corticosteroids (73.91%; 95% CI: 53.53%-87.45%).

Of the 23 patients, 20 (85.96%) had at least one comorbidity, and the mean number of comorbidities per patient was 1.83±1.30 (95% CI: 1.26-2.39). The most common comorbidity among the patients was hypertension, affecting 12 (52.17%) patients. Other comorbidities commonly identified in our patients included diabetes (n=10; 43.48%), previous smoking habits (n=6; 26.09%), and obesity (n=3; 13.04%). Less frequent comorbidities included current smoking habits (n=2; 8.70%), arrhythmia (n=2; 8.70%), chronic cardiac disease (n=2; 8.70%), hepatic disease (n=1; 4.35%), psychiatric illness (n=1; 4.35%), and dyslipidemia (n=1; 4.35%). The mean Charlson score of our patients was 2.57±1.47 (95% CI: 1.93-3.20). All patients were classified as critical during the management of COVID-19 (8).

Regarding COVID-19 symptoms during the initial acute phase, 21 (91.30%) patients presented with at least one symptom. The most common symptom was cough (n=19; 82.61%), followed by fever (n=16; 69.57%), and dyspnea (n=16; 69.57%). Eight (34.78%) patients complained of muscle pain. Other symptoms included fatigue (n=6; 26.09%), odynophagia (n=4; 17.39%), headache (n=2; 8.70%), coryza (n=2; 8.70%), diarrhea (n=1; 4.35%), anosmia (n=1; 4.35%), and nausea (n=1; 4.35%).

Details about the patient population are presented in Table 1 (for continuous data) and Table 2 (for categorical data). Please note that for some data “n” is <27, as measurements could not be performed on all patients.

The mean duration of inpatient rehabilitation was 22.70±9.49 days. We repeated the measurements at the end of the treatment to evaluate the changes in our patients. The final measurements are presented in Table 3 and Table 4. After analyzing the before- and after-treatment results, we found that the results for MRC (*p*<0.01, n=27), FIM (*p*<0.0001, n=27) and FAC (*p*<0.01, n=27) improved significantly after treatment. Particularly, the results of the FAC test indicated that the patients were unable to walk independently at admission; however, half of them (n=13, 48.15%) achieved some form of independent walking by the end of treatment.

Additionally, we focused on the treatment duration and investigated possible correlations between treatment duration and test results.

**Figure 1:**
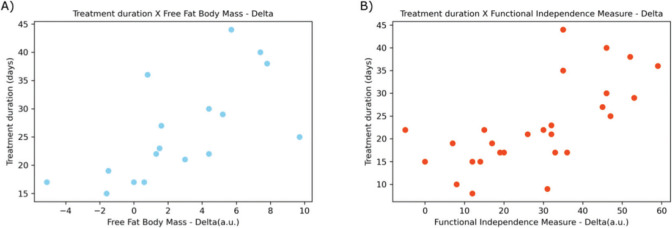
Scatterplots displaying the correlation between A) treatment duration and fat-free body mass, and B) treatment duration and Functional Independence Measure. a.u., arbitrary units.

We found a moderate positive correlation between “FIM-Delta” and treatment duration (Spearman’s r=0.71, n=27), and between FFBM-Delta and treatment duration (Spearman’s r =0.79, n=18). Positive correlations are visualized in the scatter plots presented in Figure 1. The dispersion of data points in a diagonal from the axis origin to the upper right corner is characteristic of a positive correlation between the variables analyzed, in which the variables increase in tandem. This positive correlation indicated that both scores improved with an increase in the number of treatment days. However, we were unable to establish causal relationships.

## DISCUSSION

The findings of this study indicated that the inpatient rehabilitation program helped significantly improve the patient’s muscle strength (MRC, *p*<0.01), ambulation ability (FAC, *p*<0.01), and overall functional independence (FIM, *p*<0.0001), suggesting that treatment can improve patients’ quality of life after discharge. Furthermore, we found that the treatment duration was positively correlated with the evolution of the FIM score (Spearman’s r=0.71) and the evolution of the FFBM index (Spearman’s r=0.79).

Our retrospective assessment of the effects of comprehensive, intensive, multidisciplinary, and individualized inpatient rehabilitation demonstrated a significant improvement in FIM measures before and after treatment (*p<*0.0001). The mean duration of the inpatient rehabilitation program was 22.70±9.49 days. The most important factors related to the length of inpatient rehabilitation were improvement in FIM scores and gains in lean mass. Similar to previous reports, the leading comorbidities identified in our patient population were hypertension, diabetes, obesity, and smoking (2-4). Although reports have suggested the need for prolonged rehabilitation treatment (4), neither the presence of comorbidities, age, or length of ICU stay influenced the duration of the treatment assessed in this study. In fact, greater independent walking capacity and functioning were achieved in a short, intensive inpatient program. These results were obtained in patients who were classified as critical during COVID-19 management, stayed in an ICU for 30.04±18.23 days (mean value), and needed endotracheal intubation, with a total length of hospital stay of 75±40.46 days. In our study, the levels of inflammatory markers, including PCR, was much higher than those observed in other studies (2).

Most patients were transferred from tertiary hospitals to sub-acute inpatient rehabilitation on the same day. The intensity and duration of our early inpatient rehabilitation treatment were as recommended (10), and much higher than those previously reported (3).

As expected, pulmonary function was altered in most patients for whom testing was possible. Changes in respiratory function and gas exchange may persist for years after SARS, especially in those who require ICU support and those who have associated muscle weakness (26). However, despite not reaching the expected values for age and sex, our patients showed improvements in maximal inspiratory and/or expiratory pressures (13).

Surprisingly, the majority of our patients did not present with abnormal levels of depression, anxiety, and stress. Our results are aligned with the report of a low probability of anxiety and mood disorders within 14 to 90 days of the diagnosis of COVID-19 (27). In contrast, cognitive function was altered in almost all patients, which is consistent with the reports of previous studies (9,27). Interestingly, baseline psychological and cognitive functions at admission did not influence the duration of rehabilitation interventions or the functional outcomes obtained at discharge from the rehabilitation center.

FAC, muscle strength, and the FIM scores were statistically different between measurements taken at admission and discharge. The generalized symmetrical muscle weakness observed in our patients is a common consequence of hospitalization, especially in those who require care in ICUs. Muscle weakness is caused by a combination of factors including immobility, post-intensive care myopathy and polyneuropathy, nutritional status, medications used, and other health conditions (3,4,9,26). Patients with malnutrition and protein deficiency, those administered steroids and/or neuromuscular blockers, and those presenting with comorbidities, including obesity, pressure ulcers, and diabetes, are more likely to develop post-intensive care myopathies (9,26). We argue that rehabilitation needs are higher in patients whose muscle weakness is associated with poor nutritional status. It was our initial impression that these patients would require prolonged and costly rehabilitation treatment for recovery (26). However, we observed that the profound and generalized decrease in muscle strength in our patients significantly reverted to normal during intensive inpatient rehabilitation. However, we acknowledge that recovery from critical illness, polyneuropathy, myopathy, and post-intensive care syndrome may require more than a year (4). Active, assisted, and monitored exercises must be performed progressively, fully based on the individual patient’s needs.

We also observed a significant improvement in the recovery of gait capacity during inpatient rehabilitation. Of the 14 (51.85%) patients admitted without a functional gait, 8 (29.63%) maintained the same status at discharge. Among those who already presented some degree of ambulation at baseline, 13 (48.15%) developed an independent gait. These results highlight the importance of early rehabilitation for functional recovery before patients are discharged home (8,26). The complexity of the clinical and functional deficits identified in our patients suggests that the transitional stay in a rehabilitation facility is key for comprehensive and integrated rehabilitation assessment and management.

Muscle strength and functional capacity are determining factors in the diagnosis of sarcopenia (28). Acute sarcopenia lasts for less than 6 months (28), and has been identified in patients with COVID-19 (29). The increased inflammation in COVID-19 may result in a catabolic state and anabolic resistance, which in turn leads to increased nutritional demand (29). Another important finding was the severity of malnutrition, even in patients with normal or high BMI values. Except one patient, all others presented with some degree of malnutrition at baseline. Other studies have also identified a high percentage of patients with malnutrition after COVID-19 and hospitalization (30,31). The generalized inflammation caused by COVID-19 is another factor involved in the nutritional status of patients after COVID-19 (29,30). We identified moderate and severe malnutrition in 95.80% of the patients. This relevant clinical finding suggests that COVID-19 survivors should be examined for their nutritional needs, apart from the analysis of BMI alone. We emphasize that BMI alone does not reflect the patient’s body composition and nutritional status. The association between obesity and sarcopenia is a recognized health condition known as sarcopenic obesity (32). Sarcopenic obesity is also associated with increased frailty and functional decline (32). In addition, the criteria for diagnosing malnutrition in adults vary according to the clinical context, following the consensus published by GLIM (24). Undernutrition or malnutrition may be caused by the compromised intake and assimilation of nutrients, secondary to anosmia, loss of taste, anorexia, and weakness of masticatory muscles (29). All these factors may contribute to malnutrition, despite a proper nutrient balance being provided during hospitalization. Therefore, we hypothesized that malnutrition may be related to the disease-associated inflammatory status, in addition to reduced food intake (29,30). We suspect that without proper nutrition and carefully monitored protein intake, as conducted in our inpatient rehabilitation treatment, muscle mass would not have been restored.

We also identified a degree of peripheral neuropathy that could be related to the post-intensive care syndrome, as our patients were in the hospital for COVID-19 management for 75±40.46 days, and most of them required assisted ventilation and tracheostomy during intensive care. Therefore, other strategies, including electrically induced muscle contraction, could not be prescribed.

Another important finding was the percentage of patients with swallowing disorders. Over 50% of our patients presented with mild or moderate dysphagia. Even though dysphagia did not significantly correlate with the duration of inpatient rehabilitation treatment, it should be properly assessed and addressed. Protein intake required to fight against severe sarcopenia requires the concomitant management of dysphagia to prevent aspiration pneumonia in patients with impaired pulmonary function due to SARS-CoV-2 infection.

Patients with SARS, including those with COVID-19, who receive endotracheal intubation may present with dysphagia at hospital discharge (33). The occurrence of dysphagia seems to be directly associated with the duration of mechanical ventilation (33). Lack of coordination between breathing and swallowing, trauma to the oropharynx and larynx, muscle weakness, laryngeal sensory deficits, and gastroesophageal reflux are possible causes of post-ICU dysphagia (33). In addition, sarcopenia and dysphagia are associated complications, as atrophy and weakness of the skeletal muscles of the head and neck may interfere with the mechanics of chewing and swallowing (34). In this case, the modified-consistency diet commonly prescribed for the management of dysphagia may not provide the appropriate energy and protein intake, predisposing to, or intensifying sarcopenia.

Although not as common, polyneuropathy in critical patients can affect the facial nerves, contributing to motor and sensory deficits that may also lead to dysphagia (35). In our patients, breathing, speech, and articulation imbalance were managed using specific speech pathology maneuvers.

Functional goals were fully achieved at discharge in 23 patients. Four patients were discharged at their own request, with goals partially met, because of some social factors associated with the total length of hospital stay and staying away their family members. Hospitalization due to critical health conditions profoundly impacts both patients and their family members, causing concerns and fears regarding their survival. Within the context of the pandemic, the burden of hospitalization is even higher because of the distancing measures in place. Many patients were unable to talk to or visit their family members for long periods of time. Even during sub-acute treatment, when life-threatening conditions are no longer present, an intensive inpatient rehabilitation program can become exhaustive and intensify the symptoms of social distancing.

The main limitations of this retrospective study were missing data on the acute condition and management, pre- and post-respiratory assessments, bioelectrical impedance, and DASS-21; Revised Impact of Events Scale; and the GLIM scores. Another important limitation was the small sample size and the lack of a control group. These should be considered when planning a prospective, observational study with a larger sample size and a control group, whenever feasible.

## CONCLUSIONS

At hospital discharge after management of acute COVID-19, patients present with significant pulmonary, physical, cognitive, nutritional, and speech pathology issues that should be properly assessed and addressed. The intensive, integrated, and coordinated multidisciplinary inpatient rehabilitation approach adopted at IMREA and IRLM significantly improved the patients’ functioning status, and could serve as a reference for the post-acute care of selected COVID-19 survivors. The main factors influencing functional recovery were gains in lean mass, muscle strength, and FIM measures.

## AUTHOR CONTRIBUTIONS

Imamura M, Mirisola AR, Ribeiro FQ contributed to the conceptualization, data curation, investigation, methodology, validation, visualization, writing the original draft of the manuscript, review, and editing. Alfieri FM and Ramos VD contributed to visualization and assisted in reviewing and editing the original draft of the manuscript. De Pretto LR contributed to data curation, formal analysis, and writing the original draft of the manuscript. Battistella LR contributed to the conceptualization, data curation, funding acquisition, methodology, project administration, resources, supervision, validation, visualization, and reviewing and editing the original draft of the manuscript. All authors revised and approved the final version of the manuscript and are accountable for its content.

## Figures and Tables

**Table 1:** Continuous variables of the baseline demographics and characteristics of the patient population at admission for inpatient rehabilitation.

Baseline Data	N	Mean	SD	Minimum	25%	Median	75%	Maximum
LoS in a COVID-19 ward (days)	23	44.96	23.00	15.00	29.50	35.00	56.00	98.00
LoS in an ICU (days)	23	30.04	18.23	5.00	16.50	24.00	38.50	76.00
LoS (COVID-19 ward+ICU) days	23	75.00	40.46	20.00	46.50	62.00	98.00	164.00
C-reactive protein (mg/L)	23	253.37	153.29	9.00	117.20	316.60	359.10	542.10
Age (years)	27	53.78	13.34	33.00	43.50	52.00	65.00	78.00
Height (m)	27	1.72	0.08	1.56	1.66	1.72	1.77	1.87
Weight (kg)	27	82.29	22.26	56.55	64.70	76.30	85.05	132.40
BMI (kg/m^2^)	27	27.57	5.80	20.00	23.65	26.30	28.59	40.80
Fat-free body mass (kg)	23	57.62	14.57	35.40	46.15	58.10	71.70	80.70
MRC	27	43.81	7.76	29.00	40.00	46.00	48.00	56.00
FIM	27	72.63	20.34	28.00	59.50	74.00	87.00	108.00
SPPB	13	5.92	1.19	4.00	5.00	6.00	7.00	8.00
MV / iP (cm H20)	13	72.31	29.48	30.00	50.00	60.00	100.00	120.00
MV / eP (cm H20)	13	63.85	23.20	40.00	73.75	100.00	117.50	135.00

N, number; SD, standard deviation; LoS, length of stay; ICU, intensive care unit; BMI, body mass index; MRC, Medical Research Council sum score; FIM, Functional Independence Measure; SPPB, Short Physical Performance Battery; MV, manovacuometer; iP, inspiratory pressure; eP, expiratory pressure.

**Table 2:** Categorical variables of the baseline demographics and characteristics of the patient population at admission for inpatient rehabilitation.

Data	n (%)
Sex	27
Female	7 (25.93%)
Male	20 (74.07%)
FAC	27
Non-functional gait / unable to walk	14 (51.85%)
Walking dependent on continuous manual contact to support body weight	3 (11.11%)
Gait dependent on intermittent or continuous manual contact	5 (18.52%)
Walking under supervision or verbal guidance	5 (18.52%)
Independent walking on level terrain, with supervision in other environments	0 (0.00%)
Independent walking anywhere, including stairs	0 (0.00%)
DASS21_Stress	22
Normal	19 (86.36%)
Mild	2 (9.09%)
Moderate	1 (4.55%)
Severe	0 (0.00%)
Extremely Severe	0 (0.00%)
DASS21_Anxiety	22
Normal	13 (59.09%)
Mild	6 (27.27%)
Moderate	3 (13.64%)
Severe	0 (0.00%)
Extremely Severe	0 (0.00%)
DASS21_Depression	22
Normal	20 (90.91%)
Mild	2 (9.09%)
Moderate	0(0.00%)
Severe	0 (0.00%)
Extremely Severe	0 (0.00%)
IES-R	22
Normal	21 (95.45%)
Abnormal	1 (4.55%)
Cognitive function (MoCA)	27
Normal	26 (96.30%)
Abnormal	1 (3.70%)
Oropharyngeal dysphagia classification	27
Normal	13 (48.15%)
Mild dysphagia	4 (14.81%)
Mild - moderate dysphagia	10 (37.04%)
GLIM	24
No malnutrition	1 (4.16%)
Moderate malnutrition	13 (54.17%)
Severe malnutrition	10 (41.67%)
PRESSURE ULCER	27
No	8 (29.63%)
Yes	19 (70.37%)
PERCENTAGE OF FAT	23
Risk of diseases associated with malnutrition	0 (0.00%)
Below average	0 (0.00%)
Average	0 (0.00%)
Above average	8 (34.78%)
Risk of diseases associated with obesity	15 (65.22%)
MLG/EST2	23
Reduced lean mass	6 (26.09%)
Normal	17 (73.91%)
FOIS	27
Oral feeding with multiple consistencies, but in need of special preparation or compensation	11 (40.74%)
Oral feeding with multiple consistencies, without the need for special preparation or compensation, but with dietary restrictions	3 (11.11%)
Oral feeding with no restrictions	13 (48.15%)

n, number; %, percentage; FAC, Functional Ambulation Categories; DASS-21, Depression Anxiety and Stress Scale; IES-R, Impact of Event Scale - Revised; MoCA, Montreal Cognitive Assessment; GLIM, Global Leadership Initiative on Malnutrition; MLG/EST2: fat-free mass/height in m^2^; FOIS, Functional Oral Intake Scale.

**Table 3:** Continuous variables of the patients’ characteristics at discharge from the inpatient rehabilitation treatment.

Data	n	Mean	SD	Minimum	25%	Median	75%	Maximum
Final Weight (kg)	27	83.68	21.67	60.40	67.55	78.50	85.65	133.50
Final BMI (kg/m^2^)	27	28.04	5.62	21.11	24.12	26.50	29.45	41.20
Final Fat-free body mass (kg)	18	58.52	14.82	33.90	50.35	53.50	72.23	84.40
Final MRC*	27	50.67	7.45	32.00	47.00	50.00	57.00	60.00
Final FIM*	27	100.67	19.61	35.00	94.50	106.00	112.50	123.00
Final SPPB	16	8.75	2.11	3.00	8.00	9.00	10.00	12.00
Final MV / iP	10	94.00	31.78	40.00	73.75	100.00	117.50	135.00
Final MV / eP	10	94.00	33.40	50.00	65.00	90.00	117.50	150.00

n, number; SD, standard deviation; BMI, body mass index; MRC, Medical Research Council sum score; FIM, Functional Independence Measure; SPPB, Short Physical Performance Battery; MV, manovacuometer; iP, inspiratory pressure; eP, expiratory pressure.

(*) indicates that the variable had a statistically significant improvement over the initial values.

**Table 4:** Categorical variables of the patients’ characteristics at discharge from the inpatient rehabilitation treatment.

Data	n (%)
Final FAC	27
Non-functional gait / unable to walk	8 (29.63%)
Walking dependent on continuous manual contact to support body weight	0 (0.00%)
Gait dependent on intermittent or continuous manual contact	1 (3.70%)
Walking under supervision or verbal guidance	5 (18.52%)
Independent walking on level terrain, with supervision in other environments	7 (25.93%)
Independent walking anywhere, including stairs	6 (22.22%)
Final Oropharyngeal dysphagia classification	27
Normal	19 (70.38%)
Mild dysphagia	4 (14.81%)
Mild - Moderate dysphagia	4 (14.81%)
Final GLIM	24
No malnutrition	11 (45.83%)
Moderate malnutrition	7 (29.17%)
Severe malnutrition	6 (25.00%)
Final fat%	18
Risk of diseases associated with malnutrition	0 (0.00%)
Below average	0 (0.00%)
Average	0 (0.00%)
Above average	6 (33.33%)
Risk of diseases associated with obesity	12 (66.67%)
Final MLG/EST2	18
Reduced lean mass	4 (22.22%)
Normal	14 (77.78%)
Final FOIS	27
Oral feeding with multiple consistencies, but in need of special preparation or compensation	6 (22.22%)
Oral feeding with multiple consistencies, without the need for special preparation or compensation, but with dietary restrictions	2 (7.41%)
Oral feeding with no restrictions	19 (70.37%)

n, number; FAC, Functional Ambulation Scores; DASS-21, Depression Anxiety and Stress Scale; IES-R, Impact of Events Scale - Revised; MoCA, Montreal Cognitive Assessment; GLIM, Global Leadership Initiative on Malnutrition; MLG/EST2: fat-free mass/height in m^2^; FOIS: Functional Oral Intake Scale.
